# Resveratrol and its Nanoparticle suppress Doxorubicin/Docetaxel-resistant anaplastic Thyroid Cancer Cells *in vitro* and *in vivo*

**DOI:** 10.7150/ntno.53844

**Published:** 2021-01-01

**Authors:** Le Xiong, Xiao-Min Lin, Jun-Hua Nie, Hai-Shan Ye, Jia Liu

**Affiliations:** 1 South China University of Technology School of Medicine, Guangzhou 510006, P.R. China.; 2 Guangzhou First People's Hospital, South China University of Technology (SCUT) School of Medicine, Guangzhou 510180, China.; 3 Liaoning Laboratory of Cancer Genomics, College of Basic Medical Sciences, Dalian Medical University, Dalian 116044, China.

**Keywords:** Resveratrol, targeted nanoparticles, anaplastic thyroid cancer, docetaxel, doxorubicin, drug resistance, tumor model

## Abstract

**Background:** Docetaxel and doxorubicin combination has been widely used in anaplastic thyroid cancer/ATC treatment but often results in serious adverse effects and drug resistance. Resveratrol effectively inhibits ATC cell proliferation *in vitro* without affecting the corresponding normal cells, while its *in vivo* anti-ATC effects especially on the ones with docetaxel/doxorubicin-resistance have not been reported due to its low bioavailability. Nanoparticles with sustained-release and cancer-targeting features may overcome this therapeutic bottleneck.

**Methods:** The resveratrol nanoparticles with sustained-release and IL-13Rα2-targeting capacities (Pep-1-PEG_3.5k_-PCL_4k_@Res) were prepared to improve the *in vivo* resveratrol bioavailability. Human THJ-16T ATC cell line was employed to establish nude mice subcutaneous transplantation model. The tumor-bearing mice were divided into four groups as Group-1, without treatment, Group-2, treated by 30 mg/kg free resveratrol, Group-3, treated by 30 mg/kg Pep-1-PEG_3.5k_-PCL_4k_@Res and Group-4, treated by 5 mg/kg docetaxel/5 mg/kg doxorubicin combination. TUNEL staining was used to detect the apoptotic cells in the tumor tissues. Docetaxel/doxorubicin resistant xenografts named as THJ-16T/R were isolated and subjected to 2D and 3D culture. The docetaxel/doxorubicin and resveratrol sensitivities of the original THJ-16T and THJ-16T/R cells were analyzed by multiple methods.

**Results:** Docetaxel/doxorubicin and Pep-1-PEG_3.5k_-PCL_4k_@Res but not free resveratrol significantly delayed tumor growth (*P* < 0.01) and caused extensive apoptosis. The mice in docetaxel/doxorubicin-treated group suffered from weight loss (> 10%) and 2/3 of them died within 3 times of treatment and the chemotherapy was stop to avoid further animal loss. One week after drug withdrawal, the subcutaneous tumors regrew and the tumor volume increased 55.28% within 14 days. The cells isolated from the regrowing tumors (THJ-16T/R) were successfully cultured under 2D and 3D condition and underwent drug treatments. Compared with THJ-16T, the death rate of docetaxel/doxorubicin-treated THJ-16T/R population was lower (39.3% vs 18.0%), which remained almost unchanged in resveratrol-treated group (45.3% vs 49.3%).

**Conclusion:** Resveratrol sustained-release targeting nanoparticles effectively inhibit *in vivo* ATC growth. Docetaxel/doxorubicin suppresses ATC xenografts but causes obvious side effects and secondary drug resistance that can be overcome by resveratrol.

## Introduction

Anaplastic thyroid cancer accounts for only 1% of all thyroid cancer cases, but it leads to more than 50% of thyroid cancer related death 1 in terms of the 6 months median survival time and 20% of the 1-year survival time 2. Although comprehensive ATC treatment including surgery, radiotherapy, chemotherapy and even molecular targeted therapy has been conducted, its therapeutic outcome remains extremely dim 3, 4. The reason is that about 70% of ATCs are diagnosed at advanced stage is that the aggressive tumor cells have invaded to surrounding tissues and spread to the distal organs 5. Therefore, for patients with metastases, systemic chemotherapies based on two or more drugs are adopted to treat ATCs 6, in which the combination of paclitaxel (paclitaxel, docetaxel) with carboplatin or doxorubicin is commonly employed 5, 7. However, clinical data show that this combined remedy causes serious adverse effects and easily induces drug resistance, making the treatment difficult to complete 7-9. Because no reliable treatment has been available for the ATCs with acquired chemotherapy resistance, the patients die very shortly of unlimited local growth and distal metastases 10. Therefore, it is in urgent need to explore new anti-ATC drug(s) with lesser side effects.

Resveratrol as a polyphenol compound mainly exists in natural plants, such as grapes and their fermentation products 11. It has been well recognized that resveratrol has a variety of biological functions including anti-inflammatory 12, 13, cardiac protection 14, anticancer 15 and DNA demethylation 16. For instance, resveratrol can induce differentiation and apoptosis of ATC cells by increasing the level of reactive oxygen species 17-20 and reverse retinoic acid resistance of ATC cells by demethylation of CRABP2 gene 16. Unlike the chemotherapuetic drugs, resveratrol at an appropriate dose has little side effects on normal thyroid tissues 21-23. Given the above evidence, we speculate that resveratrol may inhibit the primary or secondary drug-resistant ATC cells and would be an alternative way for the clinical management of ATCs. Nevertheless, the bioavailability of resveratrol is very low *in vivo* due to the quick biotransformation and elimination. To overcome this dilemma, it would be necessary to design a novel resveratrol form with sustained release and ATC-targeting properties as the resveratrol nanoparticles (Pep-1-PEG_3.5k_-PCL_4k_@Res) 24 used in current study.

To investigate the *in vivo* anti-ATC effects of docetaxel/doxorubicin and resveratrol, human ATC THJ-16T cell line was selected to establish subcutaneous transplantation model in nude mice. When docetaxel/doxorubicin resistance occurs in THJ-16T xenografts, conventional two dimensional (2D) culture and three dimensional (3D) organoid culture were conducted to observe the cellular and molecular biological effects of resveratrol on them. This is because IL-13Rα2 is frequently up-regulated in thyroid cancers and is considered as a target protein of thyroid cancer 25. IL-13Rα2-specific PEP-1, a 9 amino acid short peptide chain 26, 27 is prepared and used for constructing Pep-1-PEG_3.5k_-PCL_4k_@Res. The *in vivo* anti-ATC effects of this sustained release and tumor-targeting nanoparticle was analyzed.

## Methods

### Cell culture

Human ATC cell line THJ-16T was provided by Dr. Liu Q (Institute of Cancer Stem Cell, Dalian Medical University, as the general gifts of Mayo Foundation for Medical Education and Research) and cultured in RPMI 1640 (Gibco, Thermo Fisher Scientific, Suzhou, China) supplemented with 5% fetal bovine serum (Gibco Life Science, Grand Island, NY, USA), 100 IU/ml penicillin, and 100 μg/ml streptomycin in a humidified atmosphere of 5% CO_2_ in air at 37 °C.

### Establishment and treatment of ATC subcutaneous tumor model

**Figure 1:**
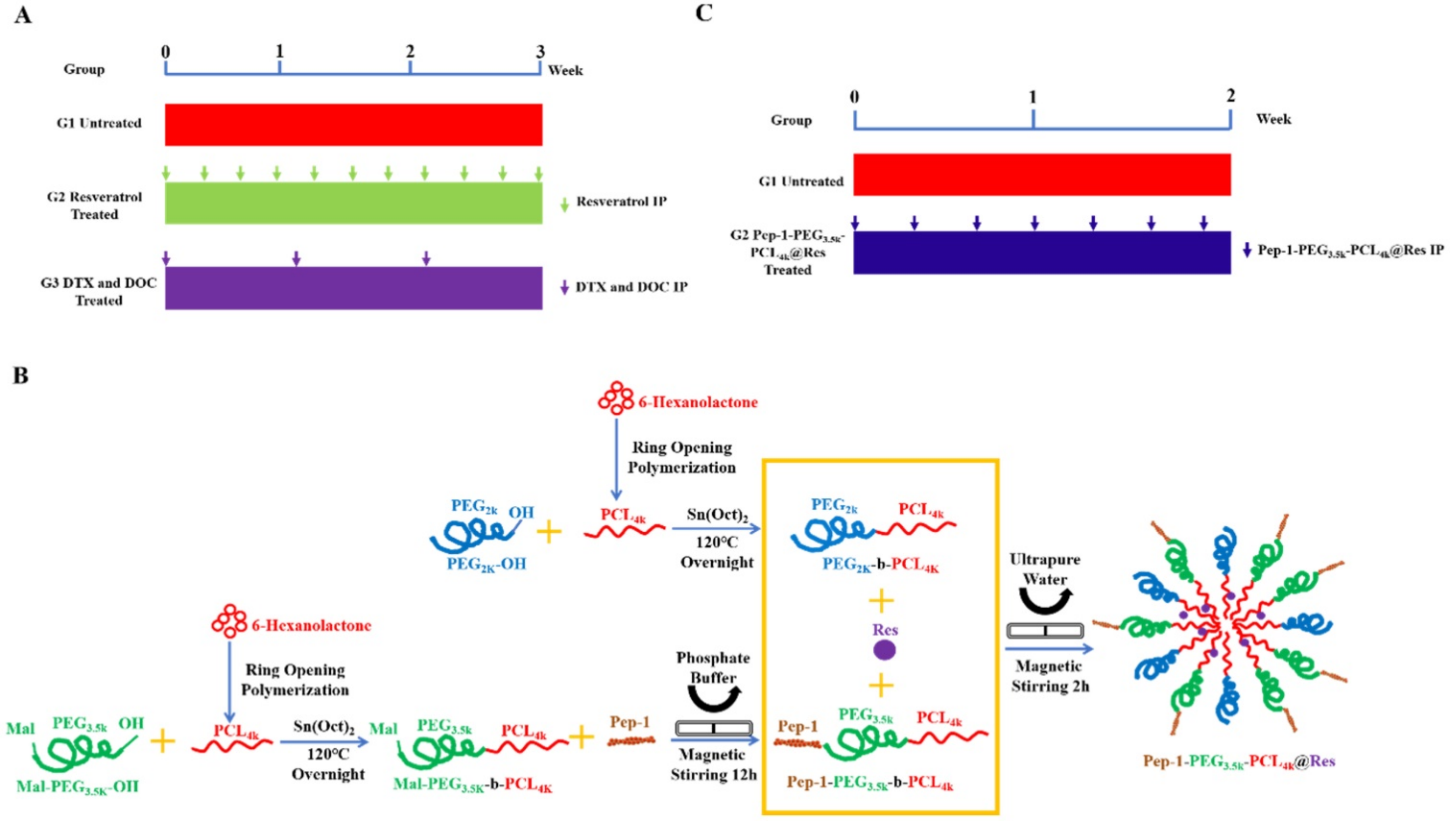
** Treatment protocol of THJ-16T subcutaneous tumor mice model.** (A) Treatment flow with free resveratrol (Res) and docetaxel (DTX) and doxorubicin (DOC) combination (CH). (B) Preparation of Pep-1-PEG_3.5k_-PCL_4k_@Res nanoparticles. (C) Treatment flow with Pep-1-PEG_3.5k_-PCL_4k_@Res.

All animal experiments were approved by animal ethics committee of South China University of Technology (AEC No. 2018050). Female BALB/c nude mice in 4 weeks were purchased from Hunan SJA laboratory animal Co., Ltd. Nude mice were reared in specific pathogen free condition with constant temperature (20-26 °C) and constant humidity (40-70%) with light/dark cycle for 12 h, and arbitrary food and drinking water. The best efforts were paid to reduce the animal suffering and control their numbers. One week after environmental adaptation, 5 × 10^6^ THJ-16T cells were subcutaneously injected into the back, and the tumors formed were passaged to the nude mice for *in vivo* treatments 28-30. The tumor-bearing animals were randomly divided into four groups as: the control group (without treatment), free resveratrol-treated group 31, 32, Pep-1-PEG_3.5k_-PCL_4k_@Res-treated group (30mg kg/day, IP), and docetaxel (5 mg/kg/week, IP) + doxorubicin (5 mg/kg/week, IP) group 33, 34. The medication flow is shown in **Figure 1A**. The tumor volumes and the body weights were measured in three-day intervals until the end of the experiment. The tumor volumes were calculated according to the formula: V = AB^2^ / 2 35, 36. V (mm^3^) is tumor volume, A (mm) is tumor length, B (mm) is tumor width. The percentage of tumor volume change = (tumor volume on the day of measurement - tumor volume at day 0) / tumor volume at day 0 * 100%, and the first day of administration is recorded as day 0 37. Euthanasia was conducted if the weight loss of tumor-bearing nude mice is close to 15% 38.

### *In vivo* Pep-1-PEG_3.5k_-PCL_4k_@Res administration

As shown in **Figure 1B** IL-13Rα2-targeting resveratrol nanoparticles (Pep-1-PEG_3.5k_-PCL_4k_@Res) were stepwise prepared by the method described elsewhere 24. Ultraviolet spectrophotometer (Evolution 300PC, Thermo Fisher, USA) was used to detect the drug concentration, and the particle size analyzer (Zetasizer Nano S90, UK) was used to evaluate the particle size. When the tumor volumes reached 50-150 mm^3^, Pep-1-PEG_3.5k_-PCL_4k_@Res in the dose of 30 mg/kg/2 days was administered intraperitoneally for 2 weeks 28-30 (**Figure 1C**). After the treatment, the tumor bearing mice were euthanized. The tumor tissues were removed and treated properly for different experimental purposes.

### 2D and 3D culture of docetaxel/doxorubicin resistant tumor cells

After withdrawing docetaxel/doxorubicin treatment, the tumors appeared rapid regrowth in time-related fashion, indicating the possible generation of their acquired drug resistance (4.70%/day increase of tumor volume during chemotherapy vs 13.00%/day increase after chemotherapy). The regrowing tumors in the docetaxel/doxorubicin-treated group were resected and cultured under conventional 2D and 3D conditions. Briefly, the resected tumor tissues were cut into small pieces and then subjected to digestion at 37 °C for 5 minutes by TrypLE (Gibco Life Science, Denmark). After centrifugation, the isolated tumor cells were suspended with DMEM/F12 medium (GIBCO, Thermo Fisher Scientific, Suzhou, China), and directly seed for 2D culture. Meanwhile, the cell resuspension was mixed with matrix gel (Corning, 356321, USA) in 2:1 ratio and then added into 48 well plate (50 μl/well) for 3D culture. After half hour incubation at 37 °C, 250 μl culture medium was added to each of the wells. The tumor-derived cell population and organoids were named as THJ-16T/R.

### Comparison of chemosensitivity of THJ-16T and THJ-16T/R

Resveratrol (Sigma Aldrich, R5010, German) and docetaxel (MCE, HY-B0011, USA) were dissolved in dimethyl sulfoxide (DMSO, Sigma Aldrich, D2650, German) into 100 mmol/L and 3 mmol/L storage solutions. Doxorubicin hydrochloride (MCE, HY-15142, USA) was dissolved in sterile distilled water into 1 mmol/L storage solution. THJ-16T and THJ-16T/R cells were treated with 0.1% DMSO as negative control, 100 μM resveratrol 13, 21, 3 μM docetaxel combined with 1 μM doxorubicin hydrochloride 39, 40, and 100 μM resveratrol combined with chemotherapy, respectively. After 24 hours of treatment, the cell-bearing coverslips were collected and underwent HE morphological staining (Keygen Biotech, Suzhou, China). Meanwhile, the cells of experimental groups were harvested and stained with trypan blue viable: unviable cell discrimination by the use of Thermo Fisher Scientific (Countess II) automatic cell counter.

### Cell proliferation and apoptosis assays

EdU fluorescent labeling was performed to elucidate proliferative activity of THJ-16T/R cells and organoids. Deoxynucleotidyl transferase-mediated dUTP-biotin nick and labeling assay (TUNEL, Beyotime Biotechnology, C1086) was used to analyze apoptotic cell death. Briefly, the cell-bearing coverslips and the organoids of each of the experimental groups were labeled with 5-ethynyl-2'-deoxyuridine (EdU) for 2 h, followed by 30 min incubation with click additive solution at room temperature in darkness. For apoptotic assay, the cell and organoid samples were incubated with TUNEL reaction solution at 37 °C for 60 min, and the cell nuclei were stained with Hoechst in dark for 10 min. The cell images were collected under a positive fluorescence microscope (Zeiss, Ax10 Axio, Germany).

### Protein preparation and Western blotting

The untreated THJ-16T cells were washed for three times with ice-cold phosphate-buffered saline and then lysed by RIPA buffer containing protease and phosphatase inhibitors. Protein samples (20 µg) were loaded onto 10% polyacrylamide gel electrophoresis and transferred to polyvinylidene difluoride membrane. The membrane was blocked by 5% skimmed milk in Tris-buffered saline (TBS-T) for 3 hours, followed by incubated with the primary antibody (IL-13Rα2, 1:800, Proteintech, 11059-1-AP, China) overnight at 4 °C. The following day, the primary antibody was discarded, and then the membrane was washed three times by TBST, followed by 1 h incubation with horseradish peroxidase (HRP)-conjugated anti-rabbit IgG. The bands were visualized by the ECL system (Amersham Imager600, GE Healthcare Life Sciences, USA). The labeling signal was removed with a stripping buffer, and the membrane was incubated with another primary antibody (GAPDH, 1:2000, Wanleibio, WL01547, China) until all the parameters were examined.

### IL-13Rα2-oriented immunohistochemical staining

The paraffin-embedded tissue sections of two ATC patients are provided from Guangdong Provincial People's Hospital (Guangzhou, China). All experiments were conducted with the approval of the Medical Ethics Committee of South China University of Technology. IHC and ICC were performed on the paraffin-embedded tissue sections of ATC patients and cell-bearing coverslips of THJ-16T cells by the method described elsewhere 19, 41. The primary antibody used was rabbit anti-human IL-13Rα2 (1:200, Proteintech, 11059-1-AP, China).

### Statistical analyses

The obtained data were statistically analyzed by SPSS 21.0. The percentage of tumor volume change and overall survival time were compared by one-way ANOVA analysis. Cell numbers and the rates of EdU- and TUNEL-positive labeling were analyzed by independent sample t-test. The histogram shows the mean ± standard deviation (SD) of the results. Statistical significance was indicated as * if *P* < 0.05, ** if *P* < 0.01 and NS if *P* > 0.05.

## Results

### Free resveratrol failed to inhibit *in vivo* ATC growth

**Figure 2:**
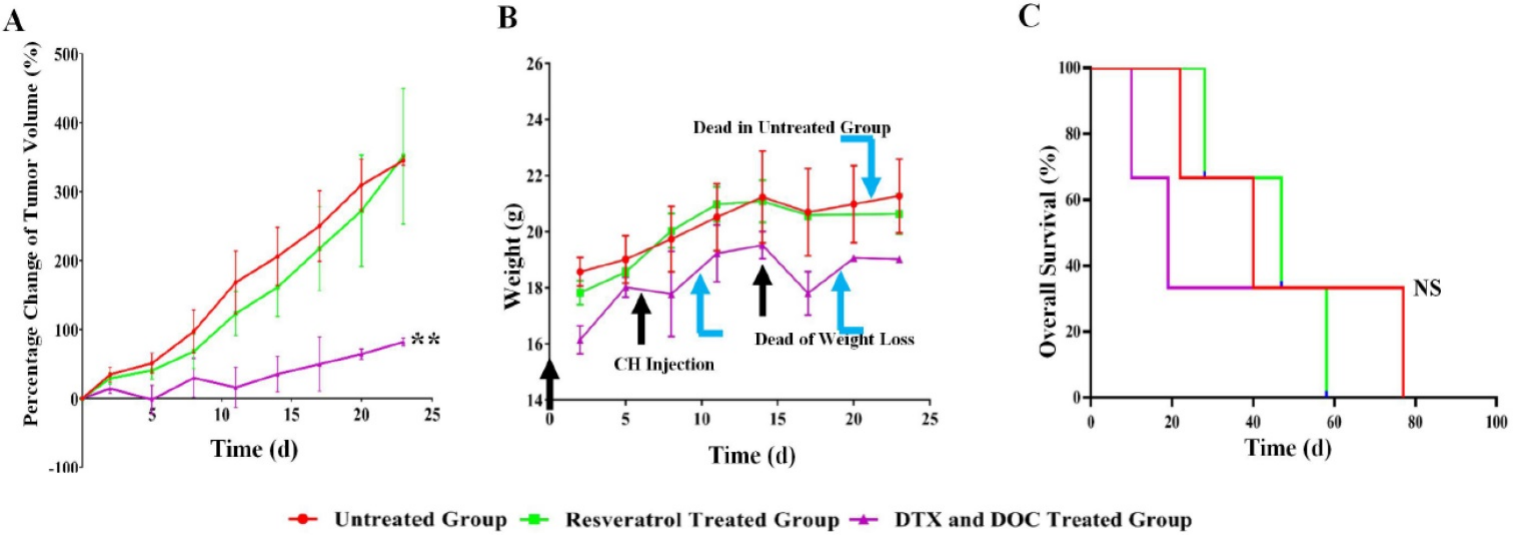
** The effects of resveratrol (Res) and docetaxel (DTX) and doxorubicin (DOC) combination (CH) on the growth of THJ-16T formed subcutaneous tumors in nude mice.** Tumor volume (A), body weight (B) and overall survival curve (C) of the experimental groups. Group-1, without treatment; Group-2, treated by free resveratrol; Group-3, treated by DTX and DOC combination. **P < 0.01 with significant difference; NS, *P* > 0.05 without statistical difference; the error bars, the mean ± standard deviation (SD).

The tumors formed by THJ-16T ATC cells (5 × 10^6^/site) were passaged to the nude mice for *in vivo* treatment and 6 transplantation tumors were prepared for each of the experimental groups. The tumor formation was observed within one week and the tumor volume reached 50 mm^3^-150 mm^3^ on the 16th day after transplantation. The tumor volumes were recorded at initial administration time (Day 0) and in 3-day intervals thereafter. The tumor growth curves were drawn according to the initial tumor volume percentage (**Figure 2A**). It was found that the average percentage of daily tumor volume change in the untreated group was 15.99% and that in the free resveratrol-treated group was 15.28%. Statistical analysis showed no significant difference between the two groups (*P* > 0.05).

### Docetaxel/doxorubicin caused tumor-suppression and severe toxicity

Compared with the growth rates of the control (15.99%) and free resveratrol-treated group (15.28%), the percentage of tumor volume increase in the docetaxel/doxorubicin-treated group was 4.70%/day in average, which was significantly slower than that of the former two groups (*P* < 0.01; **Figure 2A**). However, 2/3 of the nude mice died after three cycles of drug treatment, and the heathy condition of the remaining docetaxel/doxorubicin-treated mice was extremely poor and suffered from obvious emaciation (14.76%). The chemotherapy was therefore stopped (**Figure 2B**). Consequently, combined docetaxel/doxorubicin therapy failed to prolong overall survival time of tumor bearing nude mice in terms of 29.0 days in the docetaxel/doxorubicin-treated group, 46.3 days in the untreated group and 44.3 days in free resveratrol-treated group (*P* > 0.05; **Figure 2C**).

### Residual tumor regrowth after docetaxel/doxorubicin treatment

**Figure 3:**
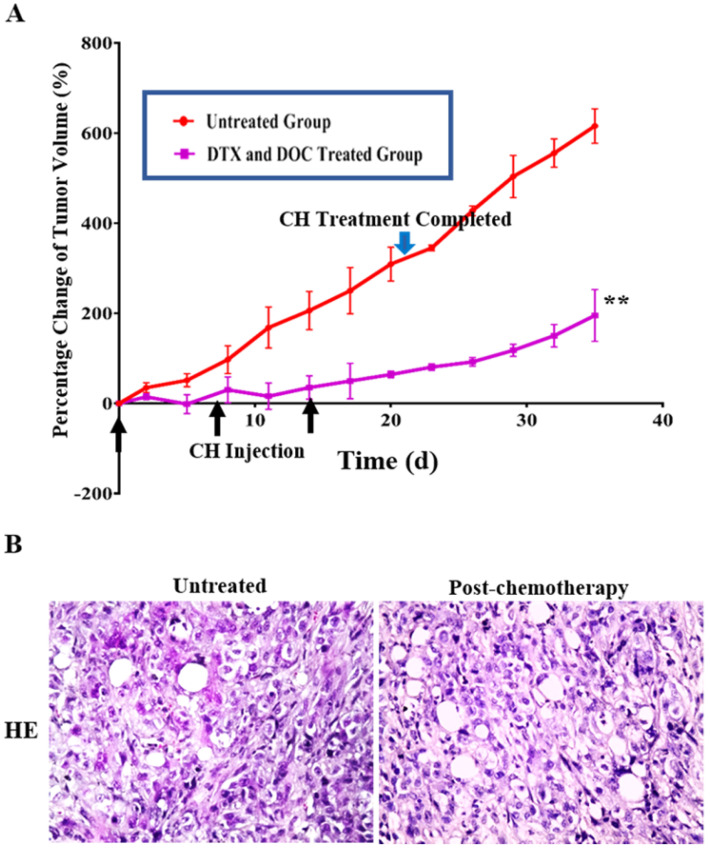
** Regrowth of THJ-16T subcutaneous tumors after withdrawing from docetaxel (DTX) and doxorubicin (DOC) treatment.** (A) Tumor volume change (%) and (B) HE histological staining (x 40) of the untreated and docetaxel/doxorubicin-treated groups during and after treatment. The black arrow means the time of CH injection and the green arrow means the time of CH administration completed. **, P < 0.01 with significant difference; the error bars, the mean ± standard deviation (SD).

The tumor bearing animals withdrawn from docetaxel/doxorubicin treatment were raised under conventional SPF conditions, and their average tumor volume showed 55.3% increase from 210.36 mm^3^ at the end of the chemotherapy to 326.65 mm^3^ within 2 weeks (**Figure 3A**). The average percentages of tumor growth during and after chemotherapy were significantly different (*P* < 0.01) in terms of 4.70%/day during and 13.00%/day after chemotherapy. HE staining confirmed that the regrowing masses were tumor tissue without necrosis (**Figure 3B**).

### Docetaxel/doxorubicin tolerance of THJ 16T/R cells

**Figure 4:**
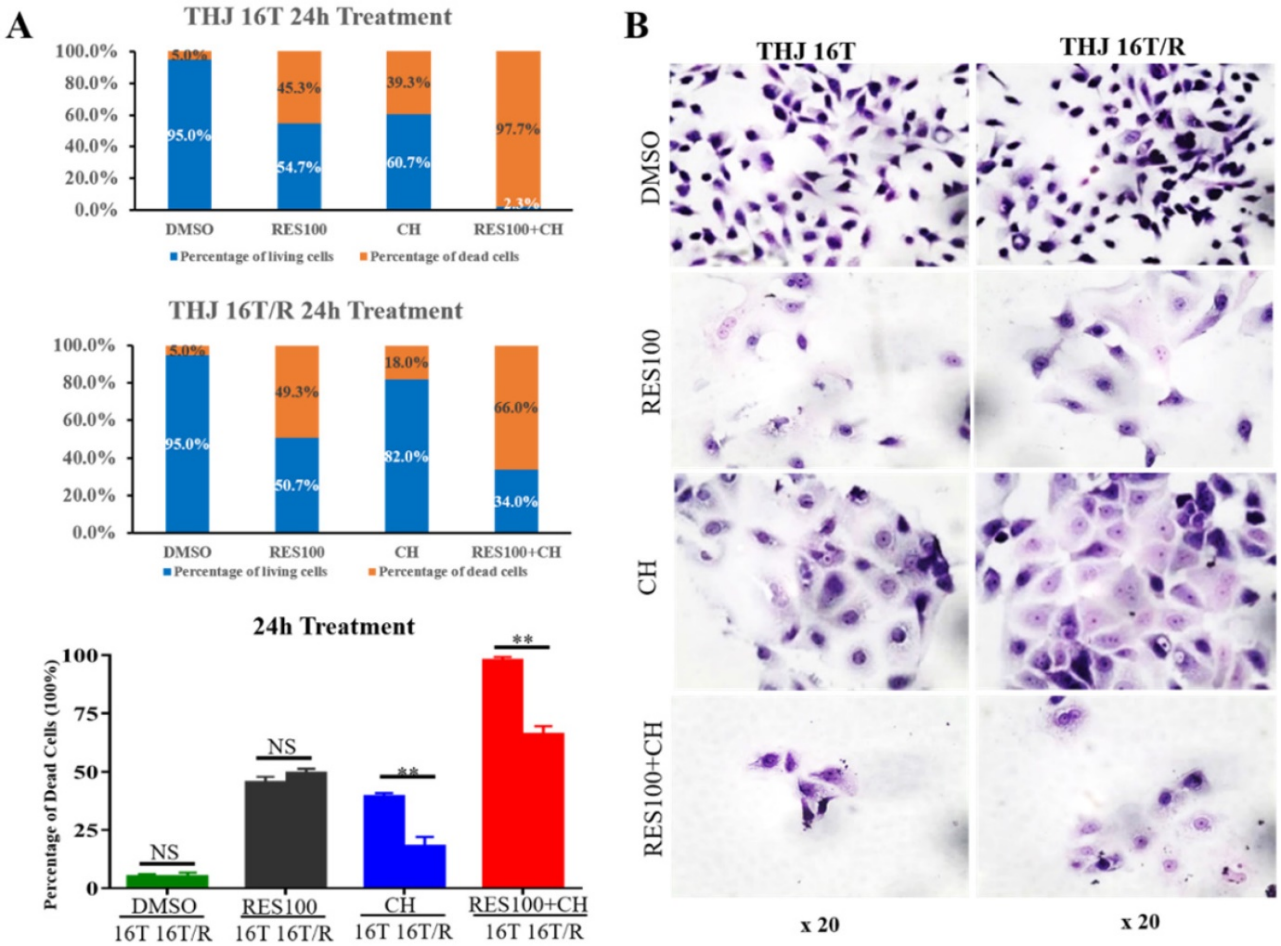
** The fractions of living and dead cells and morphology of THJ-16T and THJ-16T/R cells without and with drug treatment.** (A) The percentage of living and dead cells in THJ-16T cells and THJ-16T/R cells; (B) HE staining of THJ-16T and THJ-16T/R cells. **P < 0.01 with significant difference; NS, *P* > 0.05 without statistical difference; the error bars, the mean ± standard deviation (SD).

**Figure 5:**
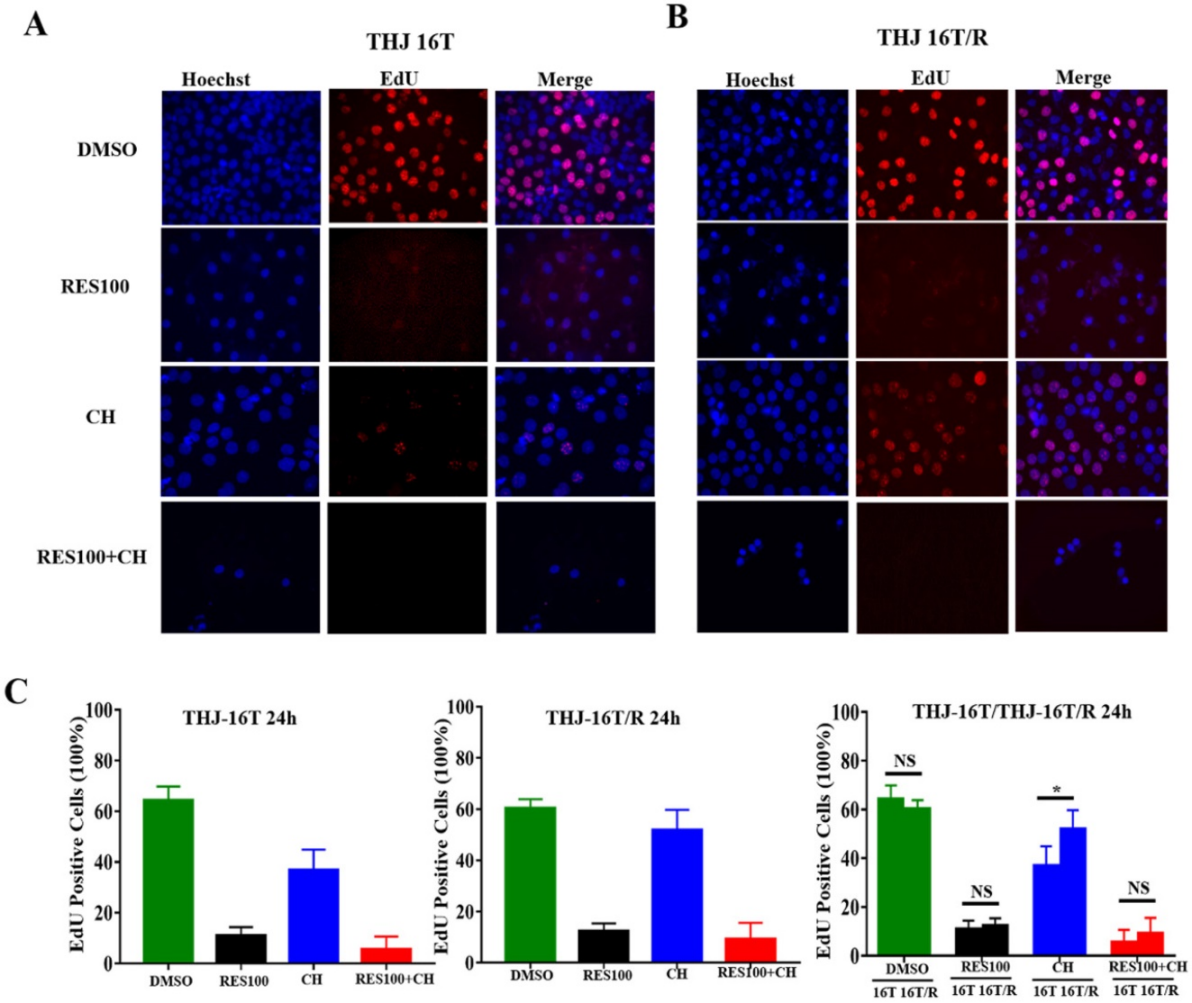
** Evaluation of proliferative activities of THJ-16T and THJ-16T/R cells after drug treatment.** (A) EdU labeling performed on THJ-16T and THJ-16T/R cells (x 40); (B) The percentage of EdU-positive cells in THJ-16T and THJ-16T/R cells treated by 100 μM resveratrol (Res100), DTX and DOC combination (CH), and combination of Res100 with CH. *P < 0.05 with statistical significance; NS, *P* > 0.05 without statistical significance; the error bars, the mean ± standard deviation (SD).

**Figure 6:**
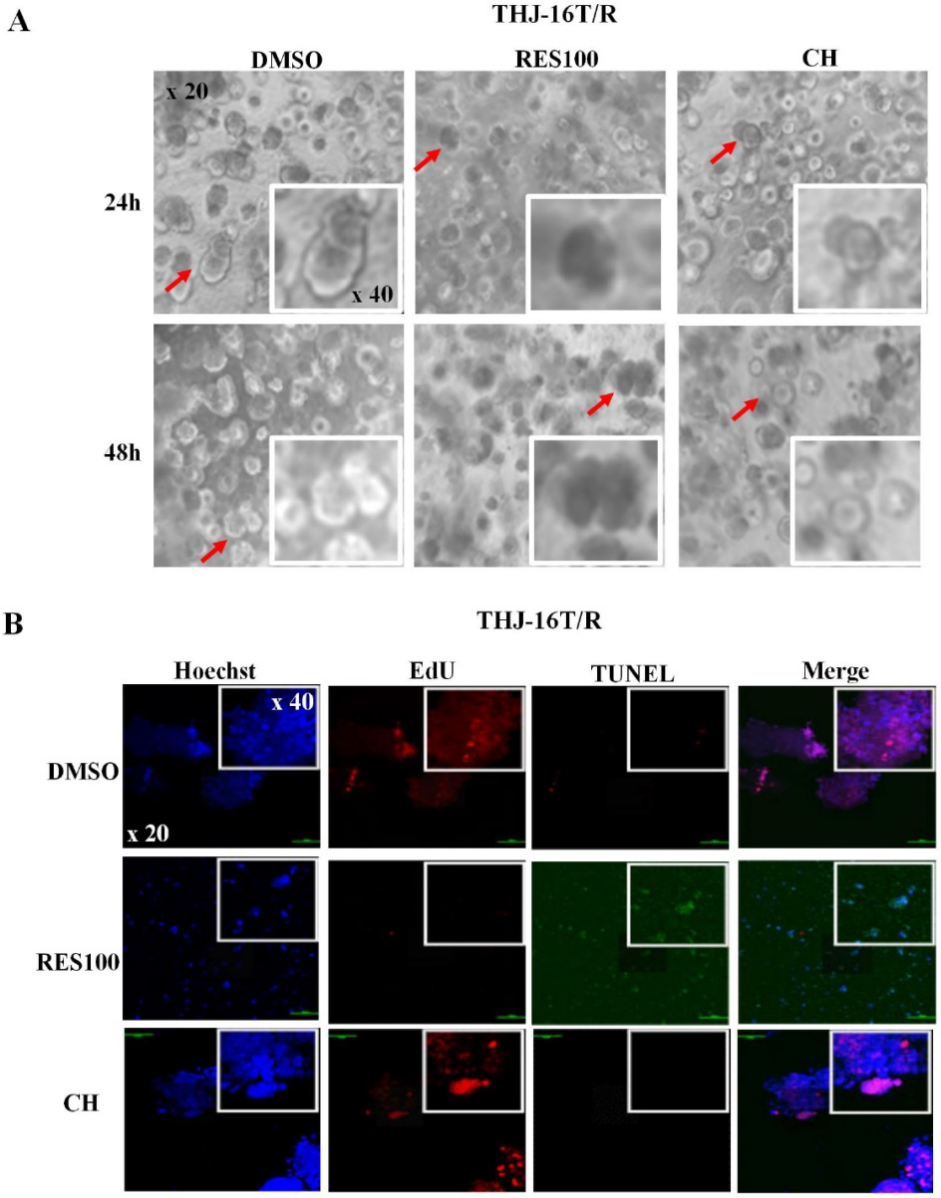
** Morphology, proliferative activities and apoptosis of THJ-16T/R-derived organoids without and with 100 µM Res or docetaxel and doxorubicin treatment.** (A) Cell morphology observed under an optical microscope (x 20); (B) EdU cell proliferation assay and TUNEL apoptotic cell labeling (x 20). Arrows indicate the portions with higher magnification (x 40) in the insets.

The cell viability assay showed that the proportion of dead cells (18.0%) in docetaxel/doxorubicin-treated THJ-16T/R group was lower than that (39.3%) of the corresponding THJ-16T group (*P* < 0.01; **Figure 4A**). HE staining revealed that THJ-16T/R shared similar morphology with its original counterpart, THJ-16T cells (**Figure 4B**). The results of EdU proliferative cell labeling showed that the percentage of EdU-positive nuclei (36.87%) in THJ-16T cells was lower than that (51.85%) in THJ-16T/R cells (*P* = 0.037; **Figure 5A-C**). After docetaxel/doxorubicin treatment, the THJ-16T/R-formed organoids remained intact with clear and bright margin (**Figure 6A**) and a large number of EdU-positive instead of TUNEL-labeled cells were observed among them (**Figure 6B**), indicating the docetaxel/doxorubicin resistant property of THJ-16T/R cells.

### Sufficient inhibitory effects of resveratrol on THJ-16T/R cells

The resveratrol sensitivity of THJ-16T/R cells were analyzed and compared with that of THJ-16T cells. Cell viability assay revealed no significant difference of the percentage of dead cells between THJ-16T and THJ-16T/R cells (*P* > 0.05), but the cell death rate of the two cell populations treated by 100 µM Res (Res100) and docetaxel/doxorubicin combination were significantly higher than that treated by Res100 (*P* < 0.01) or docetaxel/doxorubicin only (*P* < 0.01). The death rate of THJ-16T cells in the combination group was higher (97.7% vs 66.0%; **Figure 4A**). EdU labeling showed that the growth suppressive effect of Res100 plus docetaxel/doxorubicin treatment was more powerful than that of docetaxel/doxorubicin only (*P* < 0.01), but no distinct difference with that of resveratrol (*P* = 0.543; **Figure 5A-C**). In accordance, resveratrol-treated THJ-16T/R organoids showed infrequent nuclear EdU labeling, poor transparency, unclear edge and abundance of TUNEL-positive cells (**Figure 6A and 6B**).

### IL-13Rα2 upregulation in THJ-16T cells and ATC tissues

**Figure 7:**
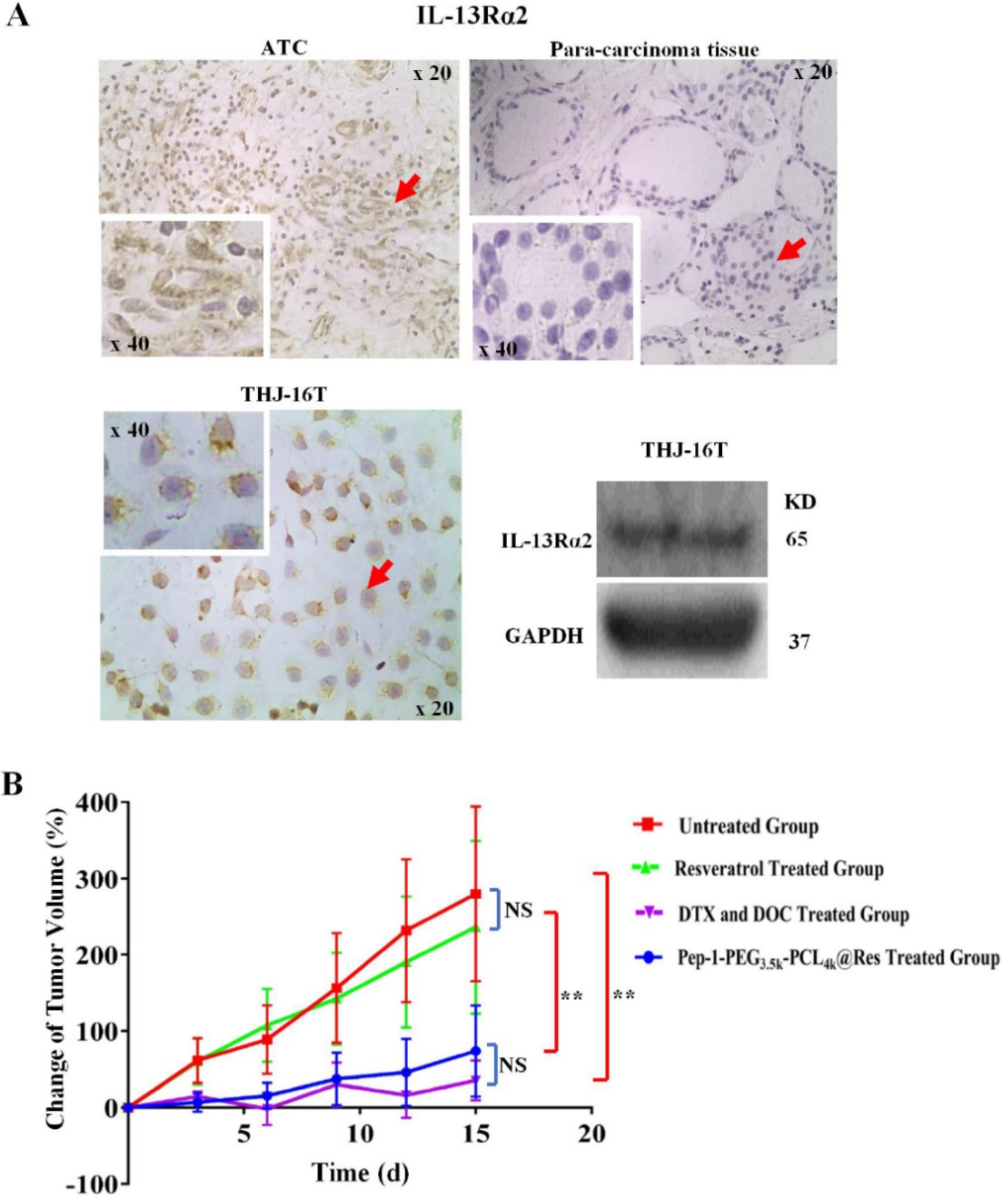
** The effects of Pep-1-PEG_3.5k_-PCL_4k_@Res on THJ-16T subcutaneous tumor model with IL-13Rα2 expression.** (A) ICC or IHC analyses IL-13Rα2 expression in THJ-16T cells and a case of anaplastic thyroid cancer and its surrounding noncancerous tissues (x 20); (B) The average volume changes (%) of the tumors treated by Pep-1-PEG_3.5k_-PCL_4k_@Res. **P < 0.01 with significant statistical difference; the error bars, the mean ± standard deviation (SD). Arrows indicate the portions with higher magnification (x 40) in the insets.

The results of immunohistochemical staining showed IL-13Rα2 expression in ATC tissue, which distributed in both the cytoplasm and cell membrane; in contrast, IL-13Rα2 was undetectable either in cytoplasm or outer membrane of normal thyroid tissue adjacent to tumor region. Western blotting results showed clear IL-13Rα2 band in the molecular weight of 65 kDa in THJ-16T cells. In accordance, immunocytochemistry showed cytoplasmic and membranous IL-13Rα2 distribution in those cells (**Figure 7A**).

### Pep-1-PEG_3.5k_-PCL_4k_@Res suppressed ATC tumor growth

**Figure 8:**
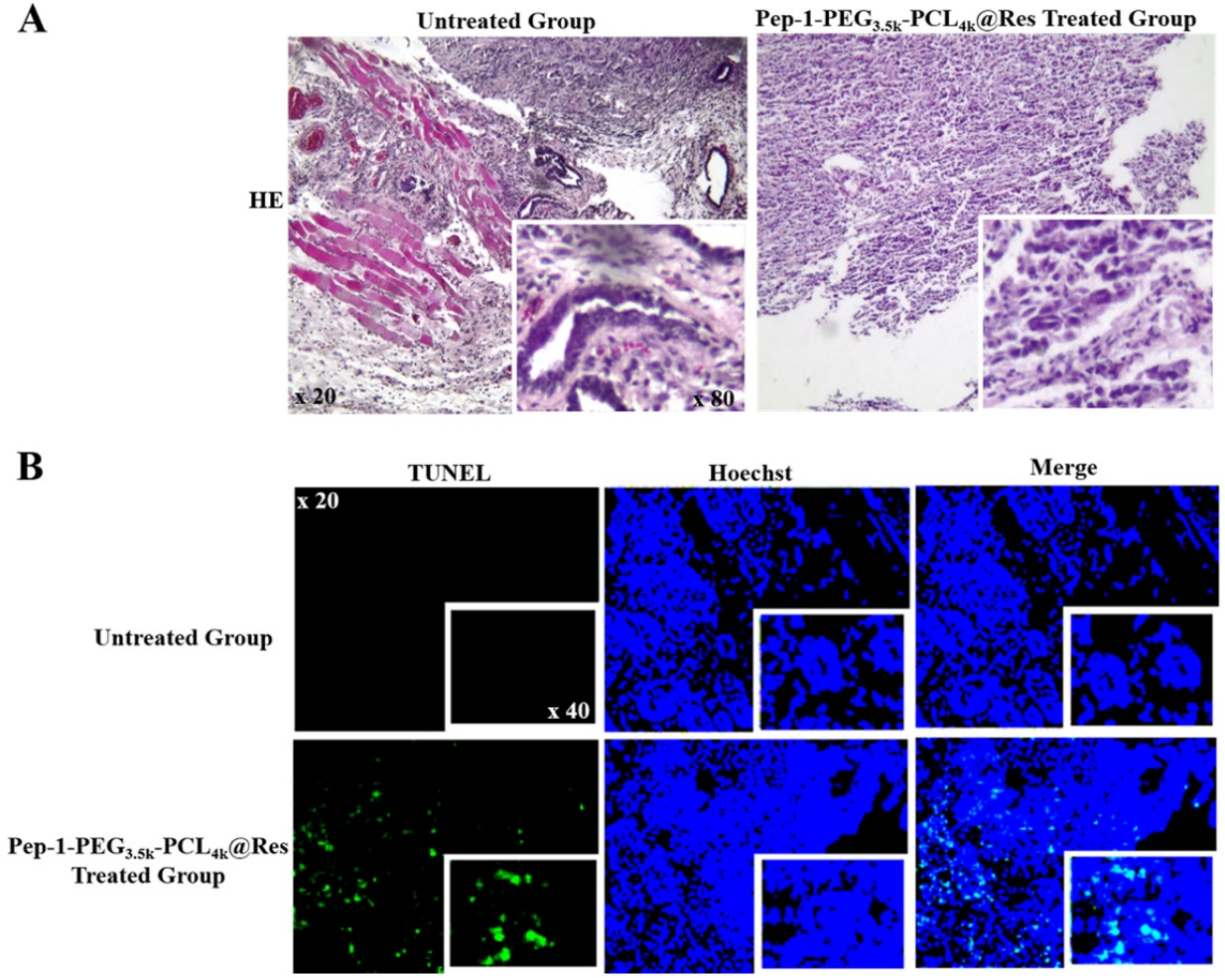
** Illustration of morphological changes and apoptosis of ATC xenografts in the untreated and Pep-1-PEG_3.5k_-PCL_4k_@Res-treated group.** HE staining (A) and TUNEL labeling (B) performed on the ATC xenografts without and with Pep-1-PEG_3.5k_-PCL_4k_@Res treatment. Inset images indicate higher magnification (x80 and x40).

It has been found that Pep-1-PEG_3.5k_-PCL_4k_@Res nanoparticle used in current study was about 30 nm in size, and its drug loading and entrapment efficiency were 6.81% and 40.84%, respectively 24. Compared with the untreated group, the tumor growth of the Pep-1-PEG_3.5k_-PCL_4k_@Res-treated group was significantly inhibited in the rate of 69.23% (4.92% vs 15.99%, *P* < 0.01; **Figure 7B**) and extensive cell death was observed in the tumor tissues (**Figure 8A**). TUNEL labeling demonstrated that apoptotic tumor cells were commonly observed in Pep-1-PEG_3.5k_-PCL_4k_@Res treated group, which were infrequent in the untreated tumors (**Figure 8B**).

## Discussion

ATC is a highly aggressive and metastatic malignancy without effective therapeutic regimen 42. Chemotherapy is still the first option for patients with metastatic ATC, while chemoresistance often occurs during repeated chemotherapy, resulting in treatment failure 6-9. Despite the use of different treatment manners, the prognosis of ATC patients remains poor, especially the ones with primary and acquired chemoresistance 4, 10. Our previous study showed that resveratrol effectively inhibited *in vitro* growth of ATC cells 19-22, and has preventive and therapeutic effect on the thyroid carcinogenesis induced by diethylnitrosamine (DEN), N-methyl-N-nitrosourea (MNU) and dihydroxydipropylnitrosamine (DHPN) 41. Although resveratrol is able to enhance chemosensitivity of the cultured cancer cells 43, 44, the impacts of resveratrol on docetaxel/doxorubicin-resistant ATC cells remain unknown. It would be therefore of clinical significance if the anti-ATC effects and the conversion ability of resveratrol to docetaxel/doxorubicin-resistance can be confirmed *in vivo*.

As the first step of current study, THJ-16T ATC cell line was employed for establishing subcutaneous transplantation tumor model in nude mice. Docetaxel combined with doxorubicin was intraperitoneally injected once a week to the tumor-bearing animals 33, 34. The results showed that this combined treatment significantly inhibited the growth of subcutaneous tumors, confirming that THJ-16T cells were sensitive to initial chemotherapy, which was consistent with previous reports 33, 45. However, this systemic chemotherapeutic approach causes severe nonspecific cytotoxicity and intolerable side effects of tumor patients, leading to the reduction of chemotherapy dose and even the interruption of treatment 46, 47. This situation reappears in current study, because 2/3 of the docetaxel/doxorubicin-treated nude mice died of serious drug toxicity only after three cycles of docetaxel/doxorubicin treatment, and the body weight of the survived animals decreased significantly, forcing the chemotherapy to be terminated. Therefore, despite the anti-ATC effect of docetaxel/doxorubicin at unit time point, it did not prolong the overall survival time as well as the life quality of the tumor bearing nude mice.

For the animal ethic consideration, we put the tumor-bearing mice withdrawn from the chemotherapy in the normal feeding environment for recovery. It was found that after drug withdrawal, the residual subcutaneous tumors in the average volume of 210.36 mm^3^ started re-growth to 326.65 mm^3^ within 2 weeks. This phenomenon indicated that the acquired drug-resistance might happen to those tumors as encountered in the clinical practice 8, 9. In order to ascertain this possibility, single cell suspension was prepared from the removed regrowing tumors, cultured under both routine 2D and 3D conditions, and then subjected to the treatments with docetaxel/doxorubicin, resveratrol and combination of docetaxel/doxorubicin with resveratrol, respectively. The results revealed the reduction of dead cells, more active cell proliferation and intact organoid structure among docetaxel/doxorubicin-treated cells and organoids. Because of the drug resistant property of the post-chemotherapeutic THJ-16T cells, we named this new cell strain as THJ-16T/R. The above results thus confirm the strong cytotoxic effects and the potential of docetaxel/doxorubicin to induce secondary drug resistance of THJ-16T and presumably the ATC tumors. It would be of clinical values to explore alternative way(s) to overcome this chemotherapeutic dilemma. In this context, THJ-16T/R cells may serve as an ideal experimental model to reach that goal.

It has been known that resveratrol can increase the sensitivity of tumor cells to other drugs besides its anticancer effects 43, 44. Resveratrol is also able to reverse the drug resistance of cancer cells 48, 49. The above evidence encouraged us to investigate whether resveratrol still kept effective to THJ-16T/R with acquired resistance to docetaxel/doxorubicin. The results showed that THJ-16T/R cells remained sensitive to resveratrol in the extents as similar as that of its parent THJ-16T cells and with little difference to THJ-16T/R cells treated by docetaxel/doxorubicin and resveratrol combination. These findings thus demonstrate, for the first time, the ability of resveratrol to suppress ATC cells with acquired resistance to the first-line anticancer drugs. In view of the fact that drug resistance is inevitable in ATC patients after repeated chemotherapy, the efficient suppression of THJ-16T/R cells by resveratrol may undoubtedly provide a potential way for the clinical treatment of ATC patients with primary and secondary chemoresistance. It would be more meaningful should the anti-ATC efficacy can be proved *in vivo*.

A body of evidence reveals the anticancer effects of resveratrol on a wide range of human malignancies *in vitro* 15, 50-53. However, the corresponding *in vivo* data remain limited because trans-resveratrol can be efficiently metabolized to the less effective cis-resveratrol 54 and quickly eliminated from the body when it is administered systemically 55, 56. Consequently, it is difficult for resveratrol to suppress tumor growth in extremely low concentration 19. The same problem is encountered in current study as the repeated intraperitoneal administration of free trans-resveratrol exerts neither inhibitory effect on the THJ-16T-formed subcutaneous tumors nor the toxic effects on the treated nude mice 21, 22. Although interventional therapy may increase resveratrol bioavailability in the tumors of the target organs and therefore achieve better tumor suppressive outcome 57, it is not suitable for ATCs because of the lack of direct administration route to thyroid glands. Given the above tricky situations, it would be necessary to modify resveratrol into the form with sustained release and tumor targeting properties. For this reason, nano drug delivery system was employed here as the carrier to encapsulate resveratrol. By this way, resveratrol can be protected from being metabolized by intrinsic biological enzymes 58 and released slowly to neutral environment *in vivo* 59. Human IL-13Rα2, a 380 amino acid glycoprotein located on the plasma membrane, activates tumor related signaling molecules such as PI3K, ATK and SRC to promote tumor progression 60, 61. IL-13Rα2 is frequently up-regulated in thyroid cancer tissues and is considered as a therapeutic target of thyroid cancer 25. Therefore, the IL-13Rα2-targeting and sustain release nanoparticles (Pep-1-PEG_3.5k_-PCL_4k_@Res) were successfully prepared and were used to treat THJ-16T-formed subcutaneous tumors in the same dose and administration manner of free resveratrol. The results clearly revealed that unlike the findings from free resveratrol-treated group, Pep-1-PEG_3.5k_-PCL_4k_@Res exhibited distinct inhibitory effects on the tumors in terms of reduced growth rates and extensive apoptosis; meanwhile, in difference with the unfortunate fate of docetaxel/doxorubicin-treated animals, the general condition of the tumor-bearing mice was well maintained without animal death and weight loss during the treatment. These results thus demonstrate that the *in vivo* anti-ATC efficacy of resveratrol can be significantly improved in the form of Pep-1-PEG_3.5k_-PCL_4k_@Res. Because this drug delivery system largely overcomes the tricky problem of the low bioavailability of systemically administered resveratrol, it may offer a potential option for better management of ATCs. To further strengthen this notion, it would be necessary to demonstrate the *in vivo* anti-ATC effects of Pep-1-PEG_3.5k_-PCL_4k_@Res on ATC patient-derived xenografts (PDXs) that share similar phenotypic features to those of the primary tumors.

## Conclusion

This study demonstrates that docetaxel/doxorubicin can inhibit the growth of ATC *in vivo*, but causes severe side effects and acquired drug resistance. Resveratrol effectively inhibits the growth of docetaxel/doxorubicin resistant ATC cells, indicating its clinical value in the treatment of ATCs, especially those with chemoresistance. Sustained-release and ATC-targeting resveratrol nanoparticles Pep-1-PEG_3.5k_-PCL_4k_@Res significantly improve the anti-ATC effects *in vivo*, providing a potential way to use resveratrol in clinical treatment of ATCs as well as other kind of cancers. THJ-16T/R chemoresistant strain would be of values in probing the causes underlying the acquired drug resistance via next generation sequencing.
